# Unlocking the Alkaloid Biological Potential of Chili Pepper (*Capsicum* spp.), Cacao (*Theobroma cacao* L.), and Coffee (*Coffea* spp.) Byproducts: Characterization, Non-Conventional Extraction, Applications, and Future Perspectives

**DOI:** 10.3390/molecules30183795

**Published:** 2025-09-18

**Authors:** Anahí Cárdenas, Luis Mojica, Luis Coronado-Cáceres, Gustavo A. Castillo-Herrera

**Affiliations:** Centro de Investigación y Asistencia en Tecnología y Diseño del Estado de Jalisco, Tecnología Alimentaria, Camino al Arenero #1227, Col. El Bajío Arenal, Zapopan 45019, Jalisco, Mexico; ancardenas_al@ciatej.edu.mx (A.C.); lmojica@ciatej.mx (L.M.); lucoronado@ciatej.mx (L.C.-C.)

**Keywords:** secondary metabolites, bioactive alkaloids, capsaicin, theobromine, caffeine, non-conventional extraction

## Abstract

Chili peppers (*Capsicum* spp.), cacao (*Theobroma cacao* L.), and coffee (*Coffea* spp.) are important crops worldwide. Nearly 35%, 80%, and 45% of the respective fruits are underutilized or discarded, representing a considerable economic loss. This work reviews and analyzes the environmental factors that influence the concentration of the main alkaloids in these crops, including capsaicin, theobromine, and caffeine. Their reported anti-inflammatory, cardioprotective, neuroprotective, and cytotoxic properties are also reviewed. This work explores strategies for the revalorization of these crops, comparing alkaloid extraction methods that use non-conventional techniques, including supercritical fluid extraction (SFE), ultrasound-assisted extraction (UAE), high-pressure and -temperature extraction (HPTE), pressurized liquid extraction (PLE), pressurized hot water extraction (PHWE), enzyme-assisted extraction (EAE), and pulsed electric field-assisted extraction (PEFAE), and their combination to enhance the recovery of capsaicin, theobromine, and caffeine, leading to sustainable and innovative uses of these crops’ byproducts. Capsaicin, theobromine, and caffeine alkaloids are promising ingredients for the development of functional foods, cosmeceuticals, and pharmaceuticals.

## 1. Introduction

Agricultural byproducts are often called agricultural waste because they are not the main production objective [1]. The main waste generated by the fruit, vegetable, and grain industries comprises peels, stems, seeds, shells, starch, bran, husks, hull juice, and sugars [2]. Recently, research has been encouraged to gain perspective on the concept of agricultural waste and rethink it as a coproduct that still preserves the possibility of being used. The latter can be valorized as raw matter for the extraction of valuable bioactive compounds [3]. Agro-industrial byproducts have gained interest in the pharmaceutical and food processing industries since they contain carotenoids, phenolic acids, anthocyanins, flavanols, phytosterols, peptides, saponins, tannins, vitamins, and alkaloids, among other compounds, that can be recovered for the development of functional ingredients [4].

Cacao (*Theobroma cacao* L.), coffee (*Coffea* spp.), and chili pepper (*Capsicum* spp.) are among the most widely produced commodities worldwide; however, their applications extend beyond their traditional consumption. For instance, chili peppers and cacao have been used since the Pre-Hispanic times as natural remedies for the common cold, sore throat [5], indigestion, and weariness [6]. On the other hand, coffee has also been used to treat conditions such as cough, asthma, migraine, and stomach pain [7]. These applications are primarily attributed to the potential of caffeine, theobromine, and capsaicin, which are alkaloids contained in coffee, cacao, and chili peppers.

Alkaloids are secondary metabolites synthesized from amino acids in plants as a defense mechanism against their harsh environment, hence their bioactivity [8]. Theobromine and caffeine are purine alkaloids that originate from the purine nucleotide xanthine, which is then methylated to 7-methylxanthosine. Following its reduction to 7-methylxanthine, a hydrogen atom is substituted by a methyl group, forming theobromine. The methylation of theobromine results in the formation of caffeine [9]. On the other hand, capsaicin originates from two pathways, the phenylpropanoid pathway and the fatty acid metabolism. The step prior to the formation of capsaicin consists of the condensation of vanillylamine and 8-methyl-6-nonenouyl-CoA [10,11].

The extraction process is a crucial step to consider for ensuring the quality of alkaloids. Alkaloids could be incorporated into functional products; however, the cost and efficiency of the extraction method used must be assessed. Standard solid–liquid methods such as lixiviation, maceration, and Soxhlet extraction require high quantities of heat, energy, time, and solvents [12]. However, non-conventional extraction techniques have garnered interest as they reduce process time and solvent consumption, while increasing extract quality [13]. Methods such as supercritical fluid extraction (SFE), microwave-assisted extraction (MAE), ultrasound-assisted extraction (UAE), high-pressure and -temperature extraction (HPTE), pressurized liquid extraction (PLE), pressurized hot water extraction (PHWE), enzyme-assisted extraction (EAE), and pulsed electric field-assisted extraction (PEFAE) are being studied in order to apply them successfully. This review aims to collect and analyze the available literature related to the extraction methods, biological potential, and applications of caffeine, theobromine, and capsaicin.

## 2. Literature Search Strategy and Eligibility Criteria

In this review, a careful literature exploration was performed to analyze the available information on the extraction methods, biological potential, and applications of caffeine, theobromine, and capsaicin. For this review, original papers published after 2010 were considered from online databases such as PubMed (https://pubmed.ncbi.nlm.nih.gov/), Science Direct (www.sciencedirect.com), Scopus (www.scopus.com), Google Scholar (https://scholar.google.com/), Research Gate (www.researchgate.net), and MDPI (www.mdpi.com). The keywords used were “capsaicin”, “theobromine”, “caffeine”, “chili pepper”, “cacao”, “coffee”, “extraction”, “anti-inflammatory”, “anti-obesity”, “antiproliferative”, and “biological activity”. The authors chose the articles hierarchically based on their publication date, title, abstract, conclusions, and full manuscript.

## 3. Byproducts of Chili Pepper, Cocoa, and Coffee Cultivation

Sustainability is one of the primary targets to achieve in the agricultural industry. This sector generates a large amount of waste and byproducts that are not effectively used and generate economic losses and pollution [14]. In recent years, the recovery of bioactive substances from agricultural byproducts has emerged as a key area of investigation, given their potential economic value [15].

### 3.1. Chili Pepper

**Figure 1:**
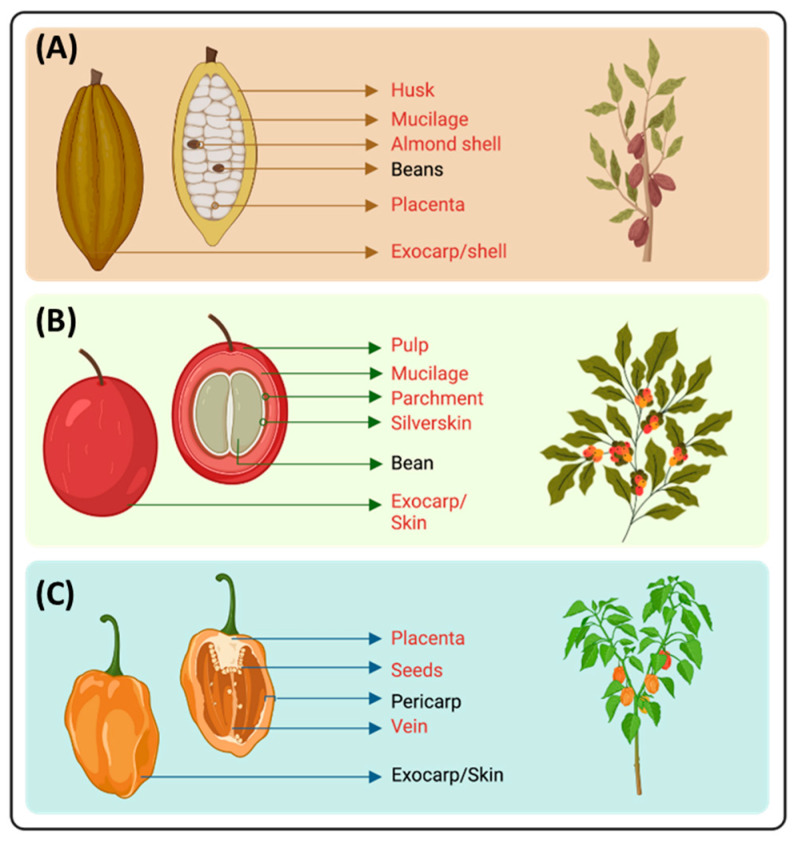
Fruit anatomy and distribution of (**A**) cacao, (**B**) coffee cherry, and (**C**) chili pepper. Red-colored words show byproducts of the fruit that are discarded in the industry.

In chillis, the pericarp is the central part consumed. Therefore, the seeds, placenta, peduncles, stems, leaves, and fruits that do not meet quality standards are discarded. These non-edible parts of chili make up 10–35% of the fruit, depending on the *Capsicum* species. Capsaicinoids are mainly accumulated in the placenta (62%); however, they are also found in the seeds (37%), pericarp (1%), leaves, and stems [16] (Figure 1).

### 3.2. Cacao

The cacao fruit (*Theobroma cacao* L.) is approximately 20 cm in length and 10 cm in diameter. The pod husk is filled with 20–40 shell-covered almonds surrounded by mucilage [17]. As displayed in Figure 1, the husk, almond shell, placenta, and mucilage are wasted byproducts [14,18].

Approximately 80% of the fruit is discarded since only the beans have industrial use [15,17], while only 10% of the fruit reaches commercialization [19]. However, cacao byproducts hold significant alkaloid content; for instance, pod husk has proved to be an abundant, inexpensive, and sustainable source of theobromine with 6.79 mg/100 g per dry weight [20]. Additionally, the cacao bean shell alkaloid content yields 9.89 mg of theobromine/g [21]. Therefore, the recovery of theobromine from cacao byproducts can be improved by incorporating the use of extraction technologies.

### 3.3. Coffee

The coffee seed grows inside the coffee tree’s fruit. After it is harvested, the skin, pulp, mucilage, silver skin, parchment, and defective beans are discarded (Figure 1), comprising approximately 45% of the fruit [22]. Additionally, the coffee tree leaves, stems, and flowers are also discarded [23,24], but caffeine is present in most of these coffee byproducts. The pulp, husk, parchment, and coffee silver skin were found to contain 0.85 g/100 g, 0.46 g/100 g, 0.06 g/100 g, and 0.71 g/100 g of caffeine, respectively, by Machado et al. [21,23]. Although the seed is the primary source of caffeine, the byproducts are still valuable sources of caffeine and several other bioactive substances, including phenolic compounds such as chlorogenic acid, caffeic acid, and ferulic acid [25].

## 4. Factors Influencing Alkaloid Content

The organoleptic properties of edible crops depend on their environmental conditions, season, species, variety, harvesting method, storage method, and drying method, if necessary [26]. Alkaloids help protect cultivars from both abiotic and biotic stress, and their levels influence organoleptic properties, such as flavor, aroma, and color [27].

### 4.1. Chili Pepper Alkaloids

**Figure 2:**
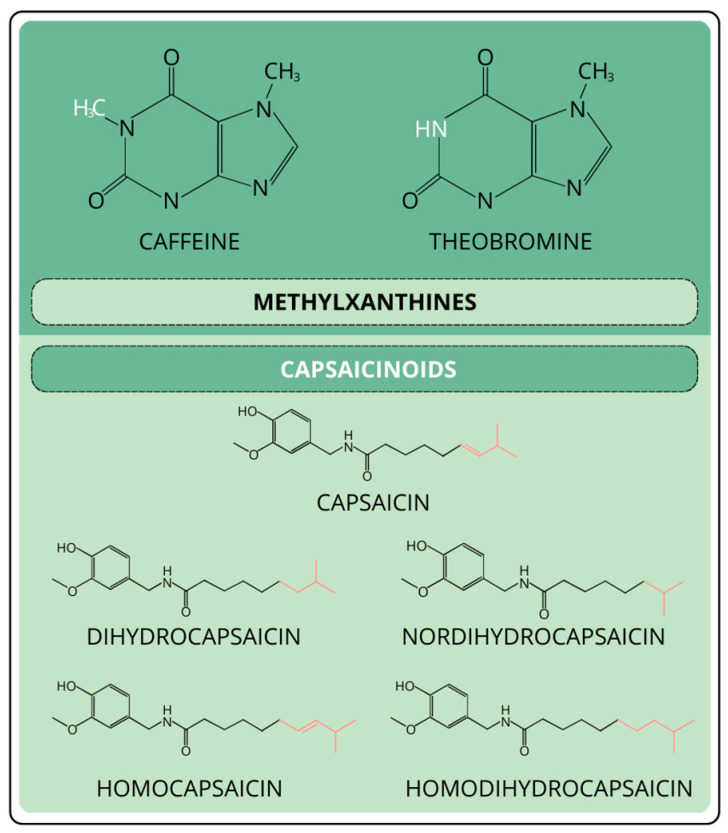
Chemical structures of theobromine, caffeine, and capsaicinoids reviewed. The different colors in the molecules indicate the functional group that changes within each set of families.

Capsaicinoids are a group of substances characterized by having a vanilloid ring in their structure. There are five main capsaicinoids found in nature: capsaicin, which is the most abundant, dihydrocapsaicin, nordihydrocapsaicin, homodihydrocapsaicin, and homocapsaicin (Figure 2) [28]. Capsaicinoid content determines the level of pungency that a certain pepper will have, and it varies depending on the plant species and the variety. Thermal stress during growth decreases the number of total capsaicinoids. The jalapeño pepper is one of the most sensitive to temperature-induced stress, where the amount of capsaicin can decrease from 21.03 mg/g dry weight to 8.11 mg/g dry weight when it grows at temperatures above 25 °C [29].

Additionally, water supply also influences the pungency level; excess humidity promotes lower pungency in the fruit. At the same time, drought conditions significantly increase capsaicinoid concentration, although it causes the fruit to have fewer seeds and a smaller size. Under stress conditions, the fruit appears to activate genes involved in the capsaicinoid synthesis pathway that occurs mainly in the placenta of the chili pepper (*Capsicum annuum* L.) [30]. Within the same variety, secondary metabolites can fluctuate depending on the geographical origin, temperature, humidity, and soil nutrients [31].

### 4.2. Cacao Alkaloids

Methylxanthines (Figure 2) are methylated derivatives of xanthine molecules produced mainly by species of tea, coffee, and cacao as secondary metabolites. The most abundant in cacao is theobromine (520–750 mg/kg), followed by caffeine (300–600 mg/kg) [32] and some traces of theophylline [33]. These molecules are responsible for the astringency and bitterness in cacao beans [34]. The different concentrations depend on the variety and the growth environment [35]. There are three main genetic groups of cacao: Criollo, Trinitario, and Forastero [36]. Studies have identified that Forastero cacao has the highest quantity of methylxanthines, followed by Trinitario and Criollo cacao [37,38,39]. Furthermore, the origin and the fermentation process also impact caffeine and theobromine content [38]. Research on the maturity stage of cacao beans indicates that caffeine and theobromine levels increase as the fruit matures. When cacao beans are fermented, the content of these alkaloids increases compared to unfermented beans. According to the investigation by Dang and Nguyen, at the ultimate ripening stage, the level of theobromine in the unfermented bean was 948.85 ± 13.99 mg/100 g dry weight. In comparison, the fermented beans contained 1088.28 ± 30.37 mg/100 g dry weight [40].

### 4.3. Coffee Alkaloids

Caffeine content is related to the sensory attributes of the coffee plant and its quality. The altitude, soil type, rainfall, temperature, soil pH, and quality factors influence the caffeine content, as well as the contents of other secondary metabolites, even in coffee from the same species and variety [41,42]. Since coffee plants grow better at high altitudes, they take longer to mature, and caffeine content is higher. A difference of 420 m above sea level (MASL) resulted in an increase in caffeine content from 1.31% to 1.51% and a corresponding increase from 2.95% to 5.05% in chlorogenic acid in the Merdacheriko variety of *Coffea arabica* [41]. Following the earlier statement, Gamonal et al. evaluated the relationship between sensory attributes, such as aroma, flavor, acidity, and balance, and the altitude at which coffee was grown, revealing that some varieties grown above 1000 MASL improve their sensory quality [43].

Bioactive compound levels are directly related to biological potential. Identifying and controlling aspects of the crop that impact alkaloid content is key to maximizing their biological potential.

## 5. Alkaloid Functions and Biological Potential

After examining how environmental conditions affect alkaloid content in chili peppers, coffee, and cacao, attention is now directed to the biological activities of capsaicin, caffeine, and theobromine (Table 1).

### 5.1. Capsaicin

Capsaicin is well known for its spicy taste, even though consuming chili pepper could be considered detrimental for the human body. Nevertheless, capsaicin has specific biological potentials as an antidiabetic, anticancer, and body weight control substance, highlighting its anti-inflammatory properties. Capsaicinoids contain a vanilloid group which is associated with the transient receptor potential vanilloid subtype 1 (TRPV1), a signal receptor that detects potential injuries and activates inflammation and pain sensation [44,45].

This receptor is distributed in peripheral and central tissues. It can also be found in hepatocytes, adipocytes, skeletal muscles, and endothelial cells [46,47]. Capsaicin functions as a TRPV1 antagonist [45], thereby reducing inflammation, which leads to various benefits, as summarized in the studies presented in Table 1. It decreases cell death by downregulating tumor necrosis factor alpha (TNF-α) at a concentration of 50 µM [48]. Additionally, by downregulating the expression of interleukins IL-2, IL-6, and IL-1β, it influences proinflammatory cytokines and decreases inflammation through the TRPV1 at 3.05 µM [49]. Capsaicin has exhibited an effect on glucose metabolism and body weight gain by increasing glucose homeostasis and reducing body weight gain in an animal model at 0.01% capsaicin in a high-fat diet (HFD) [46], processes which are linked with the antidiabetic and anti-obesity potential of this molecule. A higher dose of 5% capsaicin in an HFD mice model enhanced lipid metabolism in the liver and adipose tissue by increasing the expression of HMG-CoA reductase, CPT-1, FAT/CD36, and GLUT4, markers related to cholesterol metabolism, fatty acid oxidation, fatty acid uptake, and glucose transport, respectively [50]. Specifically, capsaicin’s antidiabetic potential has been studied carefully. Besides improving glucose metabolism, capsaicinoids also increased insulin levels and glycogen content in diabetic male Sprague Dawley rats fed 6 mg of capsaicin per kilogram of body weight [48,51]. By adjusting the dose, capsaicin can mediate various responses targeting diabetes, obesity, and other related conditions.

Inhibition of cell proliferation has also been demonstrated in Caco-2 colorectal adenocarcinoma cells and OE19 esophageal cells (IC_50_, 81.9 μM) [49], as well as in hepatocellular carcinoma (IC_50_, 172.8 μM) [50]. Recent studies have also considered the promising effect of capsaicin consumption on gut microbiota by improving functions such as tyrosine, sucrose, and starch metabolism [51]. Capsaicin from chili pepper is a substance consumed orally [47]; nevertheless, its spicy flavor limits its application in functional foods. For this purpose, analogs of capsaicin have been developed to obtain the benefits of its consumption without the taste; however, strategies for encapsulating and delivery are needed to improve and expand capsaicin applications.

### 5.2. Theobromine

Theobromine is present in a world-renowned sweet treat: chocolate. Consuming this snack has been linked with detrimental effects, mainly regarding its sugar and fat content. However, theobromine has proved to exert anti-inflammatory, antiproliferative, neuro-stimulant, and even body weight management properties (Table 1). Theobromine has gained interest, and numerous studies have been conducted to explain the mechanism behind its potential benefits. In contrast to capsaicin, theobromine has the benefit of being present in a widely consumed snack. Chocolate can be useful in designing functional foods while considering final consumer expectations.

Theobromine has demonstrated anti-inflammatory properties by downregulating the expression of inflammatory interleukin IL-1β, thereby decreasing proinflammatory mediators from the inflammatory pathway, including cyclooxygenase-2 (COX-2), prostaglandin E2 (PGE2), nitric oxide synthase (iNOS), and nitric oxide (NO). It also inhibited metalloproteinases MMP-3 and MMP-13 and suppressed the nuclear factor NF-κB, as well as the tumor necrosis factor (TNF-α) and monocyte chemoattractant protein (MCP-1), at a dose of 50 μM in an in vitro study on SW135 cells, where its effect was linked to osteoarthritis [52]. These markers are associated with several conditions and have demonstrated anti-obesity potential. A dose of 200 mg/kg body weight enhanced energy expenditure and reduced body weight gain in mice, while in adults with obesity, a dose of 450 mg/day decreased waist circumference and improved high-density cholesterol levels [53], thereby contributing to the prevention of metabolic syndrome, type 2 diabetes, gut diseases [54], and cardiovascular diseases [55].

Theobromine has also demonstrated an apoptotic effect on colorectal cancer HT-29 cells (0.673 μg/mL) [56] and colorectal Caco-2 cells (10 μM) [57], as well as cytotoxicity on lung carcinoma cells (IC_50_ 40.2 µM) and on colon cancer cells (IC_50_ 34.05 µM) [58]. Compared to capsaicin’s antiproliferative potential on colorectal Caco-2 cells, theobromine presents higher apoptotic potential. This alkaloid has also shown potential as a neuroprotective agent, as shown in blocking adenosine receptors [59].

### 5.3. Caffeine

Caffeine is the most studied alkaloid among those reviewed in the present work. This substance, which is consumed daily by millions of people, has various properties, highlighting its neuro-stimulating and anti-inflammatory potential. Caffeine is an antagonist of adenosine receptors since it has a similar structure to adenosine and can bind to its receptors. Research related to caffeine’s biological potential is shown in Table 1. Caffeine’s neuro-stimulating effect has been described as potential neurotransmitter modulation, such as of serotonin, norepinephrine, and dopamine, achieved at a dose of 30 mg/kg in a rat model of depression [60]. It has also been proven to enhance antidepressant medication response in people dealing with depression at a dose of 60 mg/day [61]. Caffeine’s anti-inflammatory potential is similar to capsaicin’s potential, shown in an LPS-induced inflammation model on RAW 264.7 cells. Results showed that caffeine reduced the expression of IL-13, IL-6, and IL-12 at a dose of 200 μM [62].

Caffeine and theobromine are commonly found together in fruits such as cacao and guarana [63], and their similarity may lead to a synergistic biological effect. Their potential has been tested by Cadoná et al. [56], analyzing the antiproliferative effect of guarana extract on HT-29 colorectal cancer cells and MCF-7 breast cancer cells, where 100 μg/mL of an extract containing 12.2 mg/g of caffeine and 6.7 mg/g of theobromine decreased cell proliferation and colony formation. Given the possible interaction between these two alkaloids, further in-depth research on their anti-inflammatory, neuroprotective, and antiproliferative activity is encouraged to determine their combined potential.

Beyond alkaloid–alkaloid interactions, synergistic effects might occur through their combination with polyphenols, terpenoids, and flavonoids. When extracting from a natural source, it is common to obtain a mixture of several substances in the extract, which can enhance its bioactive potential. For instance, the employment of cacao-derived flavonols, such as epicatechin, has been shown to downregulate diastolic blood pressure; however, their combination with the methylxanthines found in cacao, including theobromine and caffeine, has enhanced their vascular positive effects [64]. Evidence has shown that polyphenols, such as thymol, and alkaloids, such as berberine, exhibit a synergistic antimicrobial effect, enhancing their potential against *Staphylococcus aureus* [65].

The synergistic underlying mechanism can be related to the targeting of multiple steps of a regulation pathway by a range of compounds. It can also be explained by substances that do not have bioactive potential but enhance the bioavailability, solubility, or absorption of the active compounds [66].

**Table 1:** Biological potential of capsaicin, theobromine, and caffeine as determined through experimental assays.

Alkaloid	Function	Model	Assay	Dose	Result	Reference
Capsaicin	Anti-inflammatory	Myoblast cells	LPS-induced inflammation	50 µM	↓Calpain-1, ↓TNF-α, and ↓Capase-3	[48]
		RAW 264.7 cells	LPS-induced inflammation	3.05 µM	↓IL-1*β*, ↓IL-2, and ↓IL-6	[49]
	Anti-obesity	C57BL/6 mice	Body weight	HFD + 0.01%	↑Glucose homeostasis, ↓body weight gain	[46]
		Mice	RT-PCR, Western blot, body growth, and fat growth	HFD + 5% capsinoids	↓Body weight, ↓fat mass, ↑HMG-CoA reductase, ↑CPT-1, ↑FAT/CD36, and ↑GLUT4	[50]
	Antidiabetic	Type 1 diabetic male Sprague Dawley (SD) rats	Apparent digestibility determination of total sugar and biochemical index determination	6 mg/kg body weight	↑Glucose metabolism, ↑insulin level, ↑glycogen content	[51]
		Hepatocellular carcinoma HepG2	Fluorescence indicated by 2-NBDG	25–200 µM	↑Glucose consumption, dose-dependent	[67]
	Antiproliferative	Colon adenocarcinoma Caco-2 and esophageal OE19 cells	MTT assay	81.86 μM	↑Inhibition of cell proliferation by IC_50_	[68]
		Hepatocellular carcinoma HepG2	MTT assay	172.8 μM	↑Inhibition of cell proliferation by IC_50_	[69]
Theobromine	Antiproliferative	Colorectal cancer line HT-29 cells	Clonogenic assay	100 μg extract/mL	Antiproliferative effect	[56]
		Lung carcinoma epithelial A549 and human colon cancer HTC-116	MTT assay	40.20 µM and 34.05 µM, respectively (theobromine derivative T-1-NCA)	Inhibition by IC_50_	[58]
	Anti-inflammatory	SW1353 cells induced by IL-1β	RT-PCR, Western blot, ELISA, Luciferase assay, ROS, and intracellular NO measurement	50 μM	↓ROS production, ↓COX-2, ↓PGE_2_, ↓iNOS, ↓NO, ↓TNF-α, ↓MCP-1, ↓NF-κβ, ↓II-collagen degradation	[52]
		Human colorectal adenocarcinoma CaCo-2 cells induced by oxysterols	Cell death, immunoblotting, Cytokine by ELISA	10 μM	↓ IL-8, ↓MCP-1, ↓tight junction protein level decrease, ↓apoptotic activity	[57]
	Cardiovascular protection	Overweight and obese adults aged 40–55 years old	Anthropometric indices, blood pressure, lipid profile, and glycemic indices	450 mg/day	↓Waist circumference, ↓LDL-c/HDL-c, ↓TG/HDL-c	[53]
		C57BL/6 mice	Blood sampling, immunoblot analysis, RT-PCR	200 mg/kg body weight	↓Body weight gain, ↓PDE4D activity, and ↑energy expenditure	[55]
Caffeine	Antidepressant	Reserpine-induced rat model of depression	Neurotransmitter analysis	30 mg/kg	Regulated serotonin, norepinephrine, and dopamine; ↑AchE activity	[60]
	Antidepressant Anti-inflammatory	Male inpatients age 31–59 years old	Depressive symptoms measurement, Montreal Cognitive Assessment	60 mg/day	↑Enhance antidepressant medication, ↑cognitive function, ↓HPA hyperactivation	[61]
		Adult Swiss male mice	LPS-induced inflammation on vastus lateralis muscle	6 mg/kg of body weight	Attenuated LPS-induced catabolic state, ↓NLRP3, ↑adenosinergic receptors *Adora1* and *Adora 2A*	[70]
	Anti-inflammatory Neuroprotective	RAW 264.7 cells	Nitric oxide assay, cytokine assay, nitrite determination, RT-PCR, and Western blot analysis	200 μM	↓ NO production, ↓iNOS, ↓COX-2, ↓IL-6, ↓IL-3, ↓IL-12, ↓NF-κB, ↓phospho-p38MAPK i	[62]
		Human Neuroblastoma cells treated with HIV-1 Tat_1–72_	Aβ level quantification, immunoblotting, and immunoelution	200 μM	↓ Aβ levels, decreased vacuolar ATPase, and increased cathepsin D	[71]
	Neuroprotective	AlCl_3_-intoxicated male albino rats	Measurement of lipid peroxidation, reduced glutathione (GHS), nitric oxide, and AchE, Na^+^/K^+^-ATPase activity	20 mg/kg	↑AchE, ↑Na^+^/K^+^-ATPase activity, regulated TNF-α, ↓GHS,	[72]

Note: The symbols indicate a significant increase or decrease in the parameter in contrast to the control. All units are associated with the alkaloid displayed unless indicated otherwise. Abbreviations: Aβ, amyloid-β; AchE, acetylcholinesterase; ATPase, adenosine triphosphatase; COX-2, cyclooxygenase-2; CPT-1, carnitine palmitoyltransferase 1; FAT/CD36, fatty acid translocase/cluster of differentiation 36; GHS, reduced glutathione; GLUT-4, glucose transporter type 4; HDL-c, high-density lipoprotein cholesterol; HFD, high-fat diet; HMG-CoA, hydroxymethylglutaryl-Coa; HPA, hypothalamus-pituitary-adrenal; IC_50_, half-maximal inhibitory concentration; IL-1β, interleukin 1 beta; IL-2, interleukin 2; IL-3, interleukin-3; IL-6, interleukin 6; IL-8, Interleukin 8; IL-12, Interleukin 12; iNOS, Nitric Oxide Synthase; LDL-c, low-density lipoprotein-cholesterol; MCP-1, monocyte chemoattractant protein-1; MRSA, methicillin-resistant staphylococcus aureus; NF-kβ, nuclear factor kβ; NLRP3, nucleotide-binding domain, leucine-rich-containing family, pyrin-domain-containing-3; NO, nitric oxide; PDE4D, phosphodiesterase 4d; PGE2, prostaglandin E2; phospho-p38MAPK, phosphorylated-38 mitogen-activated protein kinase; ROS, reactive oxygen species; TC, total cholesterol; TG, triglyceride; TNF-α, tumor necrosis factor alpha.

## 6. Alkaloid Extraction Techniques

As described earlier, alkaloids have a diverse range of functions. The extraction technique must be appropriate to obtain the substance of interest without damaging its bioactive potential while achieving a high extraction yield. The traditional methods are detrimental to the environment as they require a high volume of organic solvents and a long extraction time. Extraction can be optimized by reducing particle size and increasing temperature, thereby improving solubility and reducing extraction time. Regardless, an elevated temperature can result in the degradation of thermolabile substances intended for recovery [73].

Therefore, greener alternatives have been developed to recover bioactive compounds effectively. These techniques are best known as non-conventional extraction methods since they are not yet widely used in the industry, even though they offer benefits like maximizing extraction yield, attenuating energy and solvent consumption, decreasing extraction time, and preventing the degradation of thermolabile compounds [74] (Table 2).

Caffeine is present in most of the coffee plant anatomy; for that reason, different extraction methods have been studied to obtain it and other bioactive compounds from coffee byproducts. UAE achieves a greater caffeine extraction ratio in the same amount of time as conventional methods, such as liquid–solid extraction. UAE utilizes sonic energy, which translates to a mechanical force capable of breaking into the cell walls of the material, facilitating compound extraction [75]. In coffee pulp, UAE at a temperature range of 348.15 K to 363.15 K and 396 W for 5.5 min was able to extract up to 206.6 mg/L of caffeine compared to 49.9 mg/L using the solid–liquid method, both of which used water as a solvent [76]. On the other hand, several applications are being developed to use spent coffee ground (SCG) waste, as it still contains substances worth recovering. SGC waste is commonly dried before processing, but the particle size is not always uniform because this byproduct originates from previous non-identical processes. The conditions in which the preceding operation is carried out will affect the outcome of the recovery process, regardless of the controlled variables of the extraction method. SFE has proved to be a great way to obtain caffeine, among other compounds, from SCGs. Temperature, pressure, and time are the primary parameters used to optimize this process. Depending on the objective, extraction efficiency can be improved by adding a co-solvent to adjust the polarity and increase selectivity. For instance, using 10% ethanol as a co-solvent under optimized conditions at 30 MPa and 333 K with SGCs resulted in a 12% recovery of caffeine from spent coffee grounds, corresponding to a composition of 3.96% molar caffeine [77]. The proportion of ethanol significantly influences extraction efficiency: for example, using 50% ethanol yielded up to 14.96% bioactive compounds from coffee pulp, whereas extraction with carbon dioxide alone achieved only 2.05%. Moreover, higher yields were associated with increased antioxidant activity, reaching a 5.5-fold enhancement [78]. When a co-solvent such as ethanol is added to the SFE process, the density of the solvent increases, which impacts the pressure of the system and the particle size, allowing for penetration into the vegetable source and resulting in better extraction [79].

HPTE is another non-conventional technique that has been applied to retrieve caffeine from SCGs. Silva et al. [12] obtained 317.0 ± 2.9 mg/L of caffeine, which is higher than UAE’s yield from coffee pulp [76]. Being different byproducts, the caffeine content in each one is different. When retrieved from a product with no previous extraction (as in the process of preparing coffee), the total extractable caffeine increases. This case is exemplified by green tea SFE, where 460 mg of caffeine/g was quantified [80] and an extraction efficiency of 100% was achieved, compared to a black tea waste extraction where no caffeine was extracted [81].

EAE, a non-conventional extraction technique, has gained interest due to its ability to enhance the release of bioactive compounds [82]. Coupled with PLE, it has proven to be an excellent method for recovering caffeine from crude guarana and guarana waste, achieving yields of up to 46.2 ± 0.14 g/100 g of extract and 43.53 ± 0.13 g/100 g of extract, respectively, using the enzyme cellulase. In contrast, a lower caffeine content is achieved without cellulase, yielding 9.20 ± 0.0 g/100 g of extract and 5.81 g/100 g of extract from crude guarana and guarana waste, respectively [83].

EAE is capable of breaking into the composition of the cell wall in fruits and seeds, enabling access to compounds such as capsaicinoids inside the cell wall. EAE of habanero chili pepper using cellulase demonstrated its capability to obtain capsaicin and phenolic compounds, although the conditions for each component differed. At 318.15 and a concentration of 250 UI/L over 150 min, 310.23 µg capsaicin/mL was obtained. A higher concentration of phenolic compounds was achieved over the same time but at 303.15 K and 2500 UI/L [84]. The different outcomes could be explained by the interactions between the enzyme and temperature, as well as the compound. Phenolic compounds are thermolabile, while alkaloids are less susceptible to temperature; thus, the extraction behavior is related to this condition. Although EAE by itself is not the most efficient extraction method, when coupled with another method, it can increase the extraction of the substance of interest, as is the case with caffeine [83].

SFE has also been applied to recover this alkaloid from habanero chili pepper. This method does not require water as it works with carbon dioxide at supercritical pressure. However, unlike caffeine, capsaicin is a nonpolar molecule; therefore, a nonpolar solvent can be added to increase extraction. For example, using 20% ethanol *w*/*w* in the process can yield 37.09 ± 0.84 mg/g extract [79].

Regardless of the extraction method, the pretreatment of the sample is crucial to consider. Chili pepper needs to be dried and milled before the process. This drying is commonly performed at high temperatures, which is not detrimental to capsaicin as it is stable at high temperatures; however, the extract may contain thermolabile phenolic compounds. An option to avoid high temperatures is to freeze-dry the sample. This method is favorable for increasing the total extractable compounds, as reported by Avilés-Betanzos et al., who compared the capsaicin content of freeze-dried and oven-dried Malagueta pepper extracts. The results show a lower concentration of capsaicin in the oven-dried peppers, attributed to the heat treatment, which decreases the alkaloid content [79]. On the other hand, particle size is directly related to the material’s superficial area, which controls mass transfer; thus, it is also linked to an increase in extraction yield [85]. This behavior corresponds to that seen in extraction from chili peppers; however, it does not apply to every matrix, as it depends on the nature of the source, whether it is oily or sticky. A decrease in particle size might not yield a better extraction [86].

Cacao’s shell is one of the main byproducts of cacao farming, containing interesting bioactive compounds. PHWE, SFE, and PEFAE have been applied to obtain theobromine and caffeine from this source. Utilizing only CO_2_ to process cacao shells in supercritical fluid conditions proved not to be efficient in recovering theobromine. In contrast, caffeine and fat can be removed with an efficiency above 90% from this source [87]. Although SFE by itself is not favorable for the recovery of theobromine, a coupled method using SFE as the first step and PLE with ethanol as the second step increases theobromine yield extraction, despite the application of PLE by itself proving not to be effective in extracting this alkaloid either [88]. Ethanol and methanol are often used to increase extraction since the theobromine affinity increases with nonpolar substances. Although PHWE uses water as the primary solvent in the process, the addition of ethanol can yield 2.06 g/100 g of theobromine from cacao bean shells [89].

Although there are several non-conventional techniques to retrieve theobromine, as well as caffeine and capsaicin, the right option depends on the source, conditions, and tools available to improve the extraction process. Nonetheless, the perks of implementing the use of non-conventional extraction methods are found in the time saved, the solvents used, and the extract quality, which are essential when working to incorporate these bioactive substances in novel and functional foods.

**Table 2:** Conventional and non-conventional extraction methods to obtain capsaicin, theobromine, and caffeine.

Raw Material	Extract	Method	Solvent	Conditions	Yield	Reference
Coffee pulp	Caffeine	UAE	Water	348.15 K–363.1 K, 396 W, 5.5 min	206.6 ± 0.1 mg/L	[76,90]
Crude guarana and guarana waste	Caffeine	Enzyme-assisted PLE	Water	323 K, pH 5.0, cellulase	46.2 ± 0.14 g/100 g extract and 43.53 ± 0.13 g/100 g extract	[83]
Spent coffee grounds	Caffeine	HPTE	Ethanol and water 50% *v*/*v*	423.15 K, 700 kPa and 60 min	31.7 ± 2.9 mg/100 mL extract	[12]
Spent coffee grounds	Caffeine and coffee oils	SFE	Carbon dioxide and ethanol	333 K, 30 MPa, 10% co-solvent	3.96% molar	[77]
Green tea	Caffeine	SFE	Carbon dioxide	333.15 K, 25 MPa, 3 h	100%	[81]
Green tea	Caffeine	SFE	CO_2_ + water	343.15 K, 30 MPa, 4 h	460.0 mg/g	[80]
**Habanero *(Campsicum chinense* var. Mayapan)**	Capsaicinoids	SFE	Ethanol 20% *w*/*w*	318.15 K, 10 MPa, 60 min	37.09 ± 0.84 mg/g extract	[79]
Trinidad Scorpion Moruga fruit	Capsaicin	PHWE	Water	473.15 K, 20 MPa, and 10 + 20 min of static extraction time	46.45 ± 0.410 mg/g dried sample	[91]
Trinidad Scorpion Moruga fruit	Capsaicinoids	Soxhlet	Methanol	2 h, 0.1 g chili, 150 mL methanol	42.875 ± 0.403 mg/g dried sample	[92]
Habanero chili pepper	Capsaicin and phenolic compounds	EAE	Enzyme solution 1:15 *w*/*v*	318.15 K, 150 min	310.23 µg/mL	[84]
Cacao shell	Theobromine and caffeine	PHWE	Water	Ethanol 15%, 363.15 K, 5 extraction cycles, 6 min static time	2.06 ± 0.06 and 0.48 ± 0.02 g/100 g	[89]
Cacao shell	Caffeine	SFE	CO_2_	41.3 MPa, 313 K, 90 min	91.80%	[87]
Cacao shell	Theobromine and caffeine	PEFAE	Ethanol and water 40% *v*/*v*	1:30 g/mL, 313.15 K, 2 h, 3 kW/cm, and 20 kJ/kg	+25% and +34%	[93]
Cocoa shell	Theobromine and caffeine	SFE + PLE	Ethanol	313.15 K, 20 MPa, 343.15 K, 10 MPa	-	[88]

Abbreviations: EAE, enzyme-assisted extraction; HPTE, high-pressure and -temperature extraction; MAE, microwave-assisted extraction; PEFAE, pulsed electric field-assisted extraction; PHWE, pressurized hot water extraction; PLE, pressurized liquid extraction; SFE, supercritical fluid extraction; UAE, ultrasound-assisted extraction.

## 7. Alkaloids: Current and Potential Applications in the Industry

Capsaicin, theobromine, and caffeine are highly valued for their properties, which offer numerous applications. These alkaloids have been utilized for their medicinal potential, and they also provide practical options for functionalizing products such as food, beverages, and cosmetics. Several ongoing studies focus on using plant extracts rich in alkaloids to drive innovation in these markets.

Capsaicin is being researched for its anti-inflammatory properties in topical treatments for pain caused by osteoarthritis [94], rheumatoid arthritis [95], and peripheral neuropathic pain [82]; its antifouling qualities for marine applications [96]; its ability to repel pests and preserve stored food [97]; and as a botanical insecticide [98]. The main area of use for capsaicin is pain relief due to its anti-inflammatory effects. Still, it is also being explored as a cosmeceutical and food ingredient in novel applications. Due to the increasing applications of capsaicin, the market value is expected to increase at a compound annual growth rate (CAGR) of 6.8%, reaching USD 484.6 million by 2032 [99].

Furthermore, extracts rich in theobromine coming from cacao and its byproducts have been recently studied for their application as a food ingredient to enhance antioxidant activity [100] and to improve nutritional content by enriching snacks [101]. Theobromine has also shown potential in skincare products for its antiaging activity [102]. Currently, the use of this alkaloid in dental care is being focused on dental bleaching [103] and for the treatment of carious lesions [104]. The ongoing investigations reveal an increasing interest in utilizing not only isolated theobromine but also extracts from fruit byproducts containing theobromine, such as cacao and guarana [63], to enhance biological potential. Research indicates that the theobromine market will reach a value of USD 112 million by 2030, with a forecast CAGR ranging between 5.1% and 68% [105,106,107].

Caffeine is used in various products, including energy drinks, cosmetics, and pharmaceuticals. Recent investigations discuss the use of coffee byproducts rich in caffeine in food formulations, as is the case of cookies enriched with coffee silverskin to provide fiber and antioxidant capacity [107], or the use of spent coffee grounds in muffins [108], as well as the use of coffee leaves in kombucha to increase the bioactive components of the beverage [109]. Emerging technologies such as nanoencapsulation or nanocarriers improve the delivery, stability, and bioavailability of caffeine while masking its bitter taste. This technique is particularly valuable in food development, such as in beverage formulations containing nanoencapsulated caffeine [110]. It has also been applied in cosmetics, where it enhances drug delivery, increases alkaloid stability, and improves loading capacity, as demonstrated in a nanostructured lipid carrier formulation for cellulite treatment [111]. However, the nanoencapsulation of bioactive compounds still faces several challenges, including ensuring delivery to the targeted site and designing a specific process to meet the substance’s requirements while also ensuring the safe consumption of the nanoparticles in terms of toxicity and ecotoxicity [112,113].

The global market size of caffeine is expected to change at a compound annual growth rate of 6.7 to 7.7%, reaching a value of USD 767.7 million by the year 2030. The interest in this alkaloid is increasing due to modern lifestyle changes, demanding boosting beverages, coffee-based drinks, and energy-fueling snacks. It is also valued in healthcare to develop pharmaceuticals aimed at weight control, as well as in the cosmetic and personal care sector [114,115,116,117].

## 8. Perspectives and Conclusions

Multiple studies have proved the biological potential of capsaicin, theobromine, and caffeine. However, their possible applications are still growing with available technologies. Non-emergent technologies are improving the quality and yield of the extracts. Furthermore, emergent technologies, such as nanoencapsulation, applied in the development of new products, enable the use of these alkaloids in the formulation of food, cosmetics, and pharmaceuticals that would otherwise be infeasible due to their taste, smell, and other unpalatable characteristics.

As part of the circular economy system, the utilization of agricultural byproducts to produce new developments is increasing. Future research should focus on the possibility of incorporating agricultural waste produced when crops are exposed to diseases as a potential source of bioactive compounds generated as part of the plants’ defense mechanisms exhibited under the stress caused by plagues. Characterization and comparison to their healthy counterparts should be carried out to assess the benefits they can offer. Besides being a source of income, this would also mitigate the economic loss caused by plagues.
